# Development and validation of a machine learning model predicting post-tonsillectomy hemorrhage

**DOI:** 10.1007/s00405-026-10344-3

**Published:** 2026-06-01

**Authors:** Anker Stubberud, Sverre Morten Zahl, Tor Åge Myklebust, Vegard Bugten

**Affiliations:** 1 Department of Otolaryngology, Helse Møre Og Romsdal Hospital Trust, Ålesund, Norway; 2 Department of Neuromedicine and Movement Science, NTNU Norwegian University of Science and Technology, Trondheim, Norway; 3 NorHead Norwegian Centre for Headache Research, Trondheim, Norway; 4 Department of Research and Innovation, Helse Møre Og Romsdal Hospital Trust, Ålesund, Norway; 5 Cancer Registry of Norway, Norwegian Institute of Public Health, Oslo, Norway; 6 Department of Otolaryngology, St. Olavs Hospital, Trondheim, Norway

**Keywords:** Tonsil, Artificial intelligence, Unsupervised learning, Decision curve analysis

## Abstract

**Purpose:**

To develop and validate machine learning models to predict post-tonsillectomy hemorrhage.

**Methods:**

This was a machine learning analysis of a cohort of patients included in the Norwegian tonsil registry in Norway from 03.01.2017 to 01.05.2025. A perioperative assessment was used to describe the type of surgery performed, the surgical technique used, and the methods used to achieve hemostasis. Postoperative outcomes were assessed in questionnaires 30 days after surgery. Unsupervised models were used to explore the data. Supervised models were developed to predict post-tonsillectomy hemorrhage, defined as any postoperative bleeding necessitating admission. Predictors included in the model were age, sex, type of surgery, surgical technique and means to achieve hemostasis. Model performance was evaluated with the area under the receiver operating characteristics curve (AUC). The best model was evaluated in a held-out test set. The model was explained with a SHAP plot. A decision curve analysis was conducted to assess the potential clinical utility of the model.

**Results:**

A total of 32,037 patients (mean [SD] age 17.84 [12.33]; 18,949 [59.15%] women) were included, with a mean bleeding rate of 6.17%. Unsupervised learning identified sub-groups with differences in bleeding rates. The best performing predictive model was the Adaboost classifier, achieving a test set AUC of 0.71 (95% CI 0.68–0.73). The most important predictors were middle or old age, bipolar diathermy for hemostasis and male sex. The predictive model was superior to alternative strategies in the decision curve analysis.

**Conclusion:**

Post-tonsillectomy hemorrhage could be predicted with moderate accuracy. More research is needed to assess if it has potential utility as a clinical decision-support tool.

**Supplementary Information:**

The online version contains supplementary material available at 10.1007/s00405-026-10344-3.

## Background

Tonsil surgery is a commonly performed procedure and generally considered safe [1]. One potentially serious complication is post-tonsillectomy hemorrhage (PTH) [2, 3]. Rarely, PTH can be catastrophic and even fatal [2]. In large cohorts, the incidence of PTH has been estimated to 0.8–3.2% [4, 5], but rates above 10% have also been reported [6].

Some risk factors for PTH have been identified, such as older age, male sex, surgical and hemostatic technique, and surgical method (tonsillectomy vs. tonsillotomy) [7–9]. However, it is difficult to accurately predict who will have a PTH. There is likely a complex relationship between many variables that partake in PTH. Machine learning has the potential to exploit such complex and multifaceted relationships between many variables, and has proved valuable for the prediction of a wide range of clinical outcomes across several medical disciplines [10]. To the best of our knowledge, there are only two small-scale studies that have previously evaluated machine learning for prediction of PTH [11, 12].

Further investigating the use of ML in prediction of PTH could increase our understanding of PTH, and lead to better predictions of which patients will have PTH. In turn, this could benefit both patients and the society through enabling individualized pre-emptive measures; stratification of individuals at risk, follow-up plans and point of care; and reduce the incidence, morbidity and costs associated with PTH.

The objective of this study was to develop and evaluate predictive machine learning models aiming to identify individuals at risk of PTH.

## Methods

### Study design, setting and participants

This was a predictive modeling cohort study of all individuals registered in the National Tonsil Registry in Norway from 03.01.2017 to 01.05.2025.

Since 2017, most otolaryngology clinics and surgeons in Norway have reported tonsil operations to the national Tonsil Registry (Tonsilleregisteret). In 2023, the coverage was 80.5%. After surgery, the surgeon reports the indication for surgery, the type of surgery performed, surgical technique used, methods used to achieve hemostasis, and postoperative medication use. Thirty days after surgery the patients are mailed a questionnaire where they report complications (postoperative bleeding, pain, infections), and if complications require readmission or return to theatre. The response rate to the 30-day questionnaire increased from 54% in 2019 to 83% in 2024. Six months after surgery the patients are mailed a questionnaire to assess symptom relief after surgery. During the initial years of the registry, individuals gave informed consent to participate in the register. From February 2022, all patients undergoing tonsil surgery have automatically been included in the registry, but the patients have a reservation right, which means that they can opt to not participate. The study population was defined as individuals registered in the tonsil registry between 03.01.2017 and 01.05.2025 that have not opted for exclusion and have returned the 30-day questionnaire. The data was handled anonymized with no identifiable personal data except age and sex.

The perioperative assessment and 30-day questionnaire have been validated. The perioperative assessment was demonstrated to have a near perfect inter-rater agreement (AC1 0.92 to 1.00) [13]. The 30-day questionnaire has also been demonstrated to have a high validity with AC1 interrater agreement between 0.7 and 1.00 [14]. Notably, the interrater agreement of the question assessing PTH necessitating re-admission was 0.98 (95% CI 0.90–1.0).

The regional ethics committee approved the study (REK Midt, 884,771) and granted a waiver of informed consent for participants included from February 2022.

### Data sources and data management

The features available for the machine learning analyses included patient age, sex, if the surgery was done as an outpatient or inpatient procedure, if it was a primary or repeated surgery, the indication for surgery, the surgical method, dissection technique, method to achieve hemostasis, and any postoperative bleeding necessitating action prior to discharge. The type of surgery was one-hot-encoded as tonsillectomy, adenotonsillectomy, tonsillotomy, or adenotonsillotomy. A range of different dissection techniques were reported. The most commonly used methods were encoded as separate features and included cold steel dissection, dissection with bipolar diathermy, diathermy scissors, and radiofrequency. Several other “warm” methods for dissection were also reported, but owing to the low frequency of each method, these were lumped into an “other warm” technique feature including coblation, ultracision, unipolar diathermy, laser and BiZact. Methods to achieve hemostasis were compiled into binary features including no action, only compression, infiltration of local anesthetic with adrenaline prior to dissection, bipolar diathermy, radiofrequency, other “warm” methods (coblation, laser and BiZact), and sutures/ties. Following these steps, all variables except patient age were binary and were not further preprocessed. Age was scaled with a min–max scaler on a 0–1 range to match the binary variables. Missingness was generally very low, and assumed to be completely at random. Instances with missing data were therefore omitted, rather than imputed. Instances with incomplete data, i.e. no dissection technique described, or a technique that was not classifiable were also omitted. The dataset was split in a randomized manner into training, validation, and test sets, in a 7:1:2 ratio. Partitions were kept separate and unchanged during training.

### Outcome

The outcome of interest was PTH, defined as any hemorrhage leading to re-admission to a hospital as reported by the patients in the 30-day questionnaire.

### Unsupervised learning

An unsupervised learning approach was used to explore the data. The features of the entire dataset, i.e. the combined training, validation and test sets, were included in the unsupervised model. Uniform Manifold Approximation and Projection for Dimension Reduction (UMAP) was used as a dimensionality reduction method to create a latent representation of the data in a two-dimensional space, allowing for visualization of separability of subjects, and cases and controls [15]. A grid search strategy was used to explore the effect of hyperparameter settings to identify the latent representation with the most clearly intelligible clusters. Next, hierarchical density-based spatial clustering of applications with noise (HDBSCAN) method was applied to the two-dimensional UMAP embeddings to identify data-driven subgroups in the data. The identified subgroups were described with summary statistics.

### Predictive modelling

Several standard machine learning architectures were evaluated, including Logistic Regression, Stochastic gradient descent, ridge classifier, multinomial naïve bayes, Gaussian naïve bayes, support vector machines, K-nearest neighbors, decision trees, random forest, gradient boosting, adaboost, extreme gradient boosting and light gradient boosting. Owing to the class imbalance (approximately 6:94), a series of strategies were evaluated to address imbalance, including oversampling, SMOTE, ADASYN and setting learning parameters to “class weight balanced” wherever available. In addition, generation of synthetic data with Forest diffusion [16] as a strategy for oversampling was evaluated. Models were trained on the train dataset and evaluated throughout the model development process by fivefold cross validation on the training set. The validation set was used to evaluate the impact of changes to the modeling pipeline such as data preprocessing, resampling strategies, and choice of classifier. For the best candidate models, hyperparameters were optimized with a fivefold cross-validated Bayesian search strategy with 50 iterations. The impact of hyperparameter tuning was evaluated in cross-validation and the validation set.

The area under the receiver operating characteristics curve (AUC) was used as the primary scoring metric. We also calculated F1 score, balanced accuracy, sensitivity, specificity, positive predictive value (PPV), and negative predictive value (NPV). The top performing model was chosen based on AUC in cross-validation and validation set performance. Only the top performing model was applied to the held-out test set to quantify out of sample performance.

Data were reported as frequencies and percentages and means with standard deviations (SD). Bootstrapping with 1000 iterations was used to derive 95% confidence intervals (CI) for the test set performance metrics.

### Decision curve analysis

To evaluate the potential clinical utility of the predictive model, we conducted a decision curve analysis as described by Vickers and Elkin [17]. Decision curve analysis is a method for evaluating the benefit of a predictive model, by calculating a “net benefit” of the prediction model in comparison to intervening on everyone or intervening on no one. As a simplification, the net benefit is the ability of a test to identify which patients do and do not have disease. The net benefit is calculated across a range of threshold probabilities, defined as the minimum probability of disease where further intervention would be warranted [18]. In the context of PTH, an applicable scenario could be at what probability of hemorrhage would an intervention, such as more active surveillance or administration of hemostatic medications, be deemed appropriate. We evaluated the net benefit of the different strategies at threshold probabilities of 5%, 10%, 15% and 20%. These thresholds were chosen because they are higher than the typical bleeding rate cited in the literature (0.8–3.5%) [4, 5], but lower than a probability where it would be unreasonable to not intervene. The top performing model was calibrated on the training set using a fivefold cross validated sigmoid regression model, to output prediction probabilities better aligning with the frequency of the outcome. Thereafter net benefit and net interventions avoided were calculated from the calibrated probabilities and plotted against the “treat all” and “treat none” strategies. Net benefit curves and net interventions avoided curves were also plotted for an alternative decision strategy by intervening based on a known risk factor, in this example, the use of bipolar diathermy for hemostasis [7].

### Model explainability

To better understand the predictive model, we calculated Shapley values and conducted a recursive feature elimination with fivefold cross-validation. The Shapley values were used to construct a SHAP summary plot allowing for visual interpretation of the magnitude and direction of prediction of each feature [19]. Recursive feature elimination is a feature selection method aiming to identify the optimal number of input features for optimal prediction performance, by iteratively removing one and one feature and evaluating the impact of the reduced feature space by fivefold cross-validation.

## Results

### Population characteristics

A total of 52,111 patients were included in the registry during the study period. Among these, 32,523 (62.4%) returned the 30-day questionnaire. After removal of subjects with incomplete and missing data, a total of 32,037 subjects were available for analysis. The mean age was 17.84 (SD = 12.33), and 18,949 (59.15%) were women. The overall rate of PTH was 6.17% (*n* = 1,859). The rates of PTH in the training, validation, and test sets were 6.38% (*n* = 1,431/22,425), 5.68% (*n* = 182/3,204), and 5.95% (*n* = 381/6,408), respectively. Table 1 shows the complete population characteristics. Supplementary Fig. 1 is a flow-chart of the study population.

**Table 1:** Population characteristics

Characteristic	Train (*n* = 22,425)	Validate (*n* = 3,204)	Test (*n* = 6,408)
Sex, female, n (%)	12,269 (55.16)	1,922 (59.99)	3,758 (58.65)
Age, years, mean (SD)	17.8 (12.35)	17.77 (12.25)	18.03 (12.31)
Inpatient procedure, n (%)	3,483 (15.53)	511 (15.95)	1,041 (16.25)
Repeat surgery, n (%)	343 (1.53)	45 (1.40)	105 (1.64)
Indication			
Obstruction, n (%)	6,836 (30.84)	989 (30.87)	1,911 (29.82)
Recurrent tonsillitis, n (%)	7,795 (34.76)	1,090 (34.02)	2,303 (35.94)
Chronic tonsillitis, n (%)	7,089 (31.61)	1,025 (31.99)	1,971 (30.76)
Peritonsillar abscess/peritonsillitis, n (%)	436 (1.94)	62 (1.94)	148 (2.31)
Systemic complication, n (%)	28 (0.12)	3 (0.09)	6 (0.09)
Other, n (%)	241 (1.07)	35 (1.09)	69 (1.08)
Quinsy tonsillectomy, n (%)	157 (0.70)	17 (0.53)	
Type of surgery			
Tonsillectomy, n (%)	14,843 (66.19)	2,114 (65.98)	4,294 (67.01)
Adenotonsillectomy, n (%)	3,022 (13.48)	432 (13.48)	883 (13.78)
Tonsillotomy, n (%)	1,136 (5.07)	163 (5.09)	303 (4.73)
Adenotonsillotomy, n (%)	3,424 (15.57)	495 (15.45)	928 (14.48)
Dissection technique			
Cold steel, n (%)	16,083 (71,72)	2,277 (71.07)	4,647 (72.52)
Bipolar, n (%)	1,279 (5.70)	173 (5.40)	366 (5.71)
Diathermy scissors, n (%)	5,059 (22.56)	708 (22.10)	1,373 (21.43)
Radiofrequency, n (%)	1,019 (4.54)	159 (4.96)	297 (4.63)
Other “warm” dissection, n (%)	595 (2.65)	89 (2.78)	146 (2.28)
Haemostasis technique			
Nothing, n (%)	432 (1.93)	52 (1.62)	103 (1.61)
Only compression, n (%)	1,880 (8.38)	269 (8.40)	561 (8.75)
Infiltration with LA + Adr prior to dissection, n (%)	7,729 (34.47)	1,104 (34.46)	2,278 (35.55)
Bipolar diathermy, n (%)	16,346 (72.89)	2,306 (71.97)	4,621 (72.11)
Radiofrequency, n (%)	556 (2.48)	92 (2.87)	184 (2.87)
Other “warm” haemostasis, n (%)	188 (0.84)	34 (1.06)	47 (0.73)
Sutures/ties, n (%)	765 (3.41)	98 (3.06)	224 (3.50)
Postoperative bleeding prior to discharge, n (%)	117 (0.52)	14 (0.44)	29 (0.45)
PTH, n (%)	1,431 (6.38)	182 (5.68)	381 (5.95)

*LA* local anesthetic; *Adr* adrenaline; *PTH* post-tonsillectomy hemorrhage

### Unsupervised learning

**Fig. 1:**
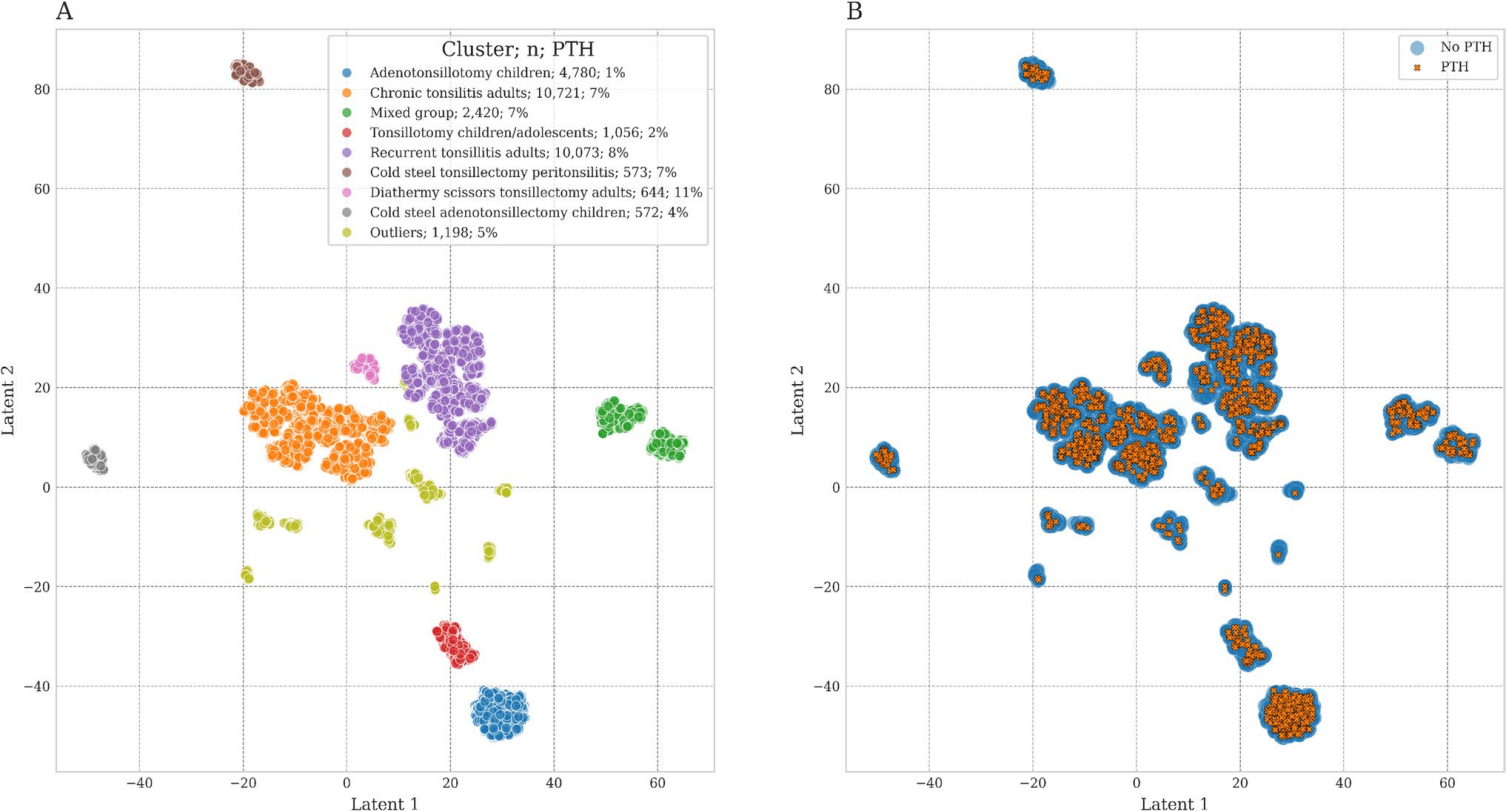
UMAP is used to create a latent representation of the dataset in two dimensions which is then plotted on a scatterplot (latent dimension 1 plotted against latent dimension 2). Panel A is colored by the HDBSCAN clustering, resulting in a total of 8 distinct clusters. The legend describes the primary characteristics of each cluster, the size of the cluster, and the PTH rate. Panel B shows the same latent representation, but here instances of no PTH are marked as a large blue dot, while instances of PTH are marked by a small orange X. UMAP: Uniform Manifold Approximation and Projection for Dimension Reduction; HDBSCAN: Hierarchical Density-Based Spatial Clustering of Applications with Noise; PTH: post-tonsillectomy hemorrhage

UMAP analysis with a hamming metric, 75 neighbors, and a minimum distance of 0.99 revealed distinct subgroups when displayed in two latent dimensions (Fig. 1). Data driven clustering with HDBSCAN corresponded to the visual UMAP representations, identifying a total of 8 clusters. Descriptive analytics of these clusters revealed distinct patterns and differences in PTH rates. Of note was that adults undergoing tonsillectomy with diathermy scissors had higher-than-average PTH rates. Children, especially when undergoing tonsillotomy or adenotonsillotomy, had lower-than-average rates of PTH. The complete descriptive statistics of the clusters can be found in Supplementary Table 1.

### Predictive model performance

The top performing model during training was the Adaboost classifier with ADASYN for resampling (tuned model hyperparameters can be found in the Supplementary Material). It achieved an AUC of 0.69 (SD = 0.01). In the test set, the classifier achieved an AUC of 0.71 (95% CI 0.68–0.73), and a balanced accuracy of 0.66 (95% CI 0.64–0.68). The complete metrics of the top model are presented in Table 2. The performance metrics of the other evaluated models during training and validation can be found in Supplementary Table 2.

**Table 2:** Performance metrics of the best predictive model (Adaboost classifier)

Metric	Training, mean (SD) of fivefold cross-validation	Test (95% CI)
AUC	0.69 (0.02)	0.71 (0.68–0.73)
Balanced accuracy	0.64 (0.02)	0.66 (0.64–0.68)
F1-score	0.67 (0.01)	0.68 (0.67–0.69)
Sensitivity	0.73 (0.04)	0.77 (0.73–0.81)
Specificity	0.55 (0.01)	0.44 (0.43–0.46)
Positive predictive value	0.10 (0.00)	0.10 (0.08–0.11)
Negative predictive value	0.97 (0.00)	0.90 (0.89–0.91)

*AUC* area under the receiver operating characteristics curve; *SD* standard deviation; *CI* confidence interval

**Fig. 2:**
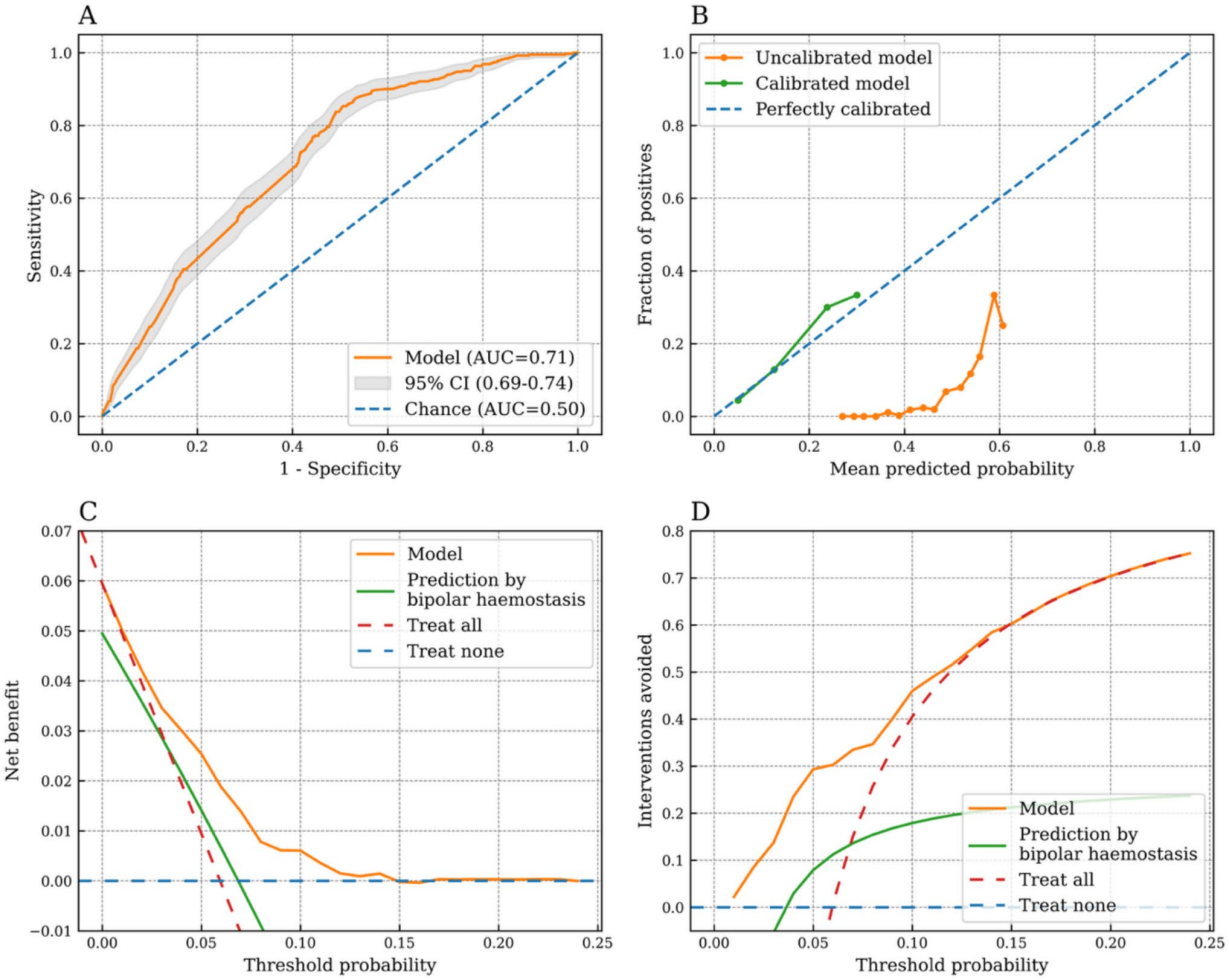
Predictive model performance and decision curve analysis. **A** Receiver operating characteristics curve of the top performing Adaboost classifier in the test set with 95% confidence interval (shaded gray area). **B** Calibration plot of the best model before (orange) and after (green) fivefold cross validated calibration. **C** Net benefit decision curve analysis plot and (D) interventions avoided decision curve analysis plot for the default “treat all” (dotted red) and “treat none” (dotted blue) strategies; as well as for the top performing predictive model (orange) and the single predictor “use of bipolar diathermy for haemostasis” (green). As shown in the decision curve analysis, the predictive model outperforms the alternative strategies above the prespecified threshold probability

Figure 2 shows the ROC-curve, calibration curve, and decision curve analysis of the best model. Calibration yielded reasonable predicted probabilities better aligning with the relatively low frequency of the outcome. In the decision curve analysis, the model outperformed both the “treat all” and the “treat none” strategies, as well as the decision to intervene based on bipolar diathermy for hemostasis. The net benefit for the predictive model was greatest at 5% and 10%, with a net benefit, as compared to treating none, of 0.03 and 0.01. The net benefit was negligible at 15% and 20%. Net benefits for all threshold for all strategies can be found in Supplementary Table 3.

### Model explainability

**Fig. 3:**
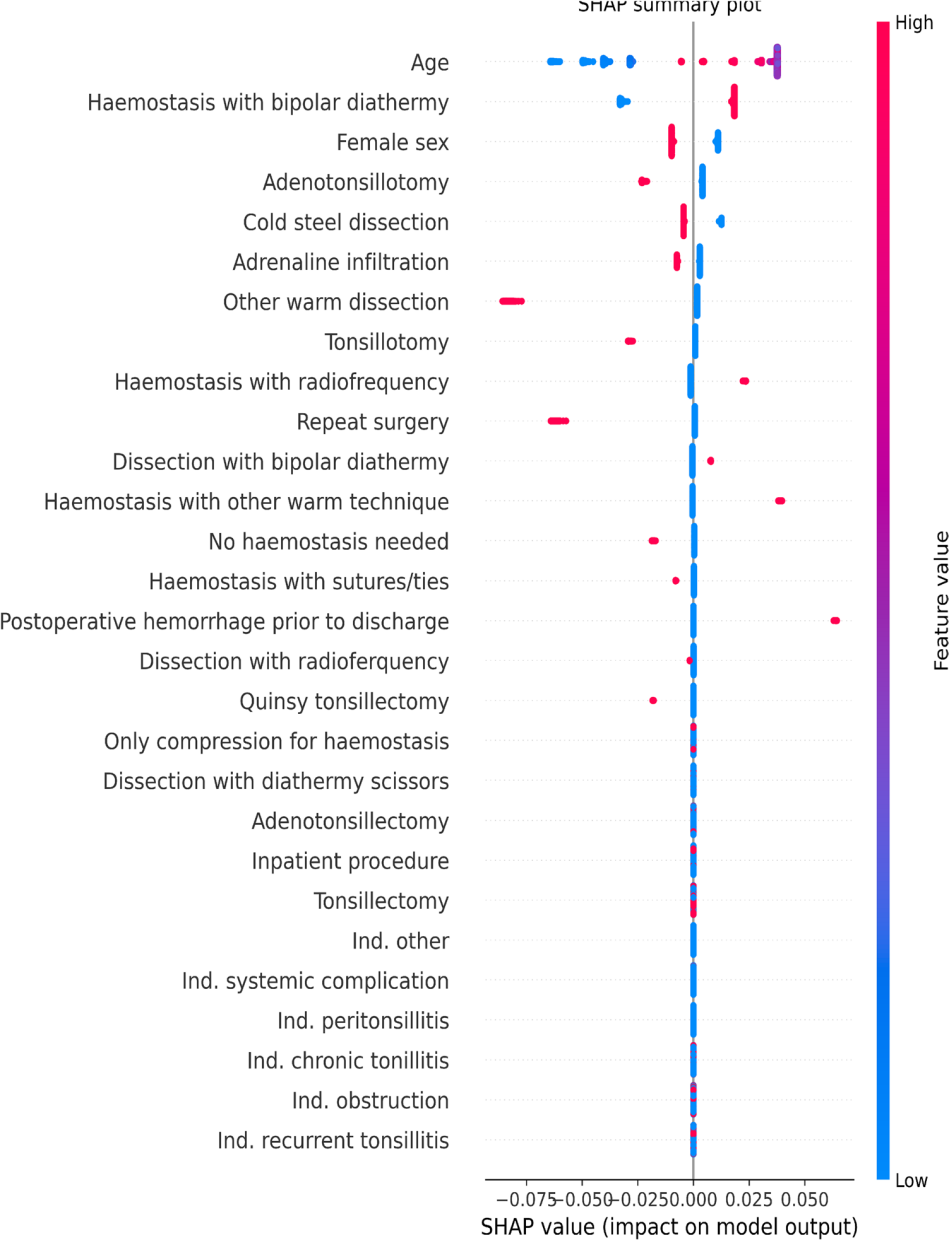
SHAP (SHapley Additive exPlanations) summary plot. The plot illustrates the impact of each feature on the model’s output. Features are ranked by their overall importance. Each point represents a Shapley value for a specific feature and individual case. Points to the right of the vertical line indicate a contribution toward predicting PTH, while points to the left indicate a contribution toward predicting no PTH. Points are colored by the feature value: for binary, red represents a positive (present) feature and blue a negative (absent) feature. For age, blue indicates younger age, and red indicates older. For example, younger age is associated with no PTH, the use of bipolar diathermy for haemostasis pushes the prediction toward PTH, and performing tonsillotomy or adenotonsillotomy pushes the prediction towards not PTH. PTH: post-tonsillectomy hemorrhage

The SHAP plot in Fig. 3 illustrates the impact of the features on the predictions. Both the SHAP analysis and the recursive feature elimination identified age, hemostasis with bipolar diathermy, sex, adenotonsillotomy, cold steel dissection, adrenaline infiltrations, other warm dissection, tonsillotomy, hemostasis with radiofrequency, primary vs. repeat surgery, dissection with bipolar diathermy, hemostasis with other warm technique, no hemostasis needed, hemostasis with sutures/ties, postoperative hemorrhage prior to discharge, dissection with radiofrequency, and quinsy tonsillectomy as important features. The optimal number of features was those seventeen, and there was a plateau in predictive performance when including additional features (Supplementary Table 4 and Supplementary Fig. 1).

All analyses were done using Python 3.11 with open source packages (Supplementary material).

## Discussion

Although the literature on machine learning in PTH is sparse, two studies are comparable to ours. A 2023 study of 520 tonsillectomies with a PTH rate of 11.54% developed a predictive XGBoost model that achieved an AUC of 0.81 [11]. A 2024 study, including 492 children undergoing tonsillectomy or tonsillotomy, also developed a XGBoost model that achieved an AUC of 0.78 [12]. Both models used demographics, comorbidities, medication use, surgical technique, and coagulation profile measurements as input data. Both of these studies outperform our model (AUC 0.71), which can have several explanations. Our model included a limited feature set of only demographics and surgical variables, while the two aforementioned studies also included comorbidities, medication use and coagulation profile measures—all potential causal contributors to PTH and likely important predictors. Although these variables were not available in our data, it is likely that their inclusion could improve predictive performance. Indeed, there are likely a wide range of unexplored potential predictors that could improve the accuracy. Future research should continue to explore the optimal set of features to be included in predictive models of PTH. That being said, the two other studies had relatively limited sample sizes, and evaluated all models across the test set, which could make them prone to overfitting and less generalizable.

Our model had a relatively high sensitivity and a very high negative predictive value, but poor specificity and poor positive predictive value. This has implications for its interpretation and clinical utility. The high sensitivity (0.77) and negative predictive value (0.90) means that the model is good at identifying those that will bleed, and that a prediction of not bleeding is correct in nine of ten cases. On the other hand, the low positive predictive value (0.10) means that nine out of ten patients predicted to bleed will not bleed. This can result in unnecessary interventions and use of resources, unnecessary patient anxiety, and limits its clinical applicability.

Because PTH is a serious and potentially fatal complication of a common surgical procedure [2, 20], the development of a prediction model is of considerable interest. However, at present, the model developed here only shows moderate accuracy, and modest benefit in the decision curve analysis. It can be interpreted as follows.

Assuming the baseline risk of PTH equals its incidence—approximately 6% in our cohort—one would expect 6 out of every 100 patients to bleed, in the absence of knowledge about the patient’s age, sex and what surgical methods were used. Next, we ask ourselves at what predicted risk would it be reasonable to intervene? I.e. at what point do we consider the risk of bleeding high enough to warrant an intervention? In the context of decision curve analysis, intervention refers to any type of action, for illustrative purposes we consider prophylactic administration of tranexamic acid.

The predicted risk threshold is visualized on the x-axis of the decision curve analysis plots as the threshold probability (Fig. 2C/D). A priori we considered risks (i.e. threshold probabilities) between 5 and 20%. To exemplify, a threshold probability of 10% corresponds to an odds of 1:9, meaning we would accept “unnecessarily” administering tranexamic acid to nine patients to prevent one PTH.

Now let’s evaluate the impact of intervening—e.g. administering prophylactic tranexamic acid—based on different decision strategies: (1) treating no patients, (2) treating all patients, (3) treating patients with increased risk according to our prediction model, and (4) treating patients exposed to a known risk factor (use of bipolar diathermy for hemostasis). As expected, treating no one yields no benefit. Treating everyone produces a benefit only when the threshold probability is lower than the prevalence. This occurs because, at the prevalence, the expected proportion of true positives equals the weighted penalty of false positives, resulting in a net benefit of zero. Our model, represented by the orange curve in Fig. 2C, slightly outperforms the alternative strategies—including the single risk factor approach—for the threshold probabilities of 5% and 10%, but is negligible at 15% and 20%.

In summary, the decision curve analysis suggest that the model provides only modest benefit for cases with moderately increased risks of bleeding (5–10%), but superior to either universal or selective intervention based on traditional risk factors (use of bipolar diathermy). At higher risk thresholds the benefit is negligible and not superior to not intervening. It is important to remember, however, that this decision curve analysis is only a mathematical framework for establishing the benefit of a predictor based on its known rate of true positives and false positives. The analysis does not take into account disease burden, side effects and cost-effectiveness, which would require a much more comprehensive decision analysis.

Our explainability analyses are mostly in line with the established risk factors for PTH. In a prospective multicenter cohort study with 9405 adults and children, those over 15 years of age were more than twice as likely to experience hemorrhage compared to children under 6 years of age [21]. In our analysis, low age was a predictor of not bleeding, while middle age was a predictor for bleeding. One could argue that age-specific models could be warranted, but we believe that when age is included as a feature in the models, the predictions are adjusted for this important risk factor. Similarly, the extensiveness of the procedure is also associated with bleeding [21]. We also found that less extensive procedures, i.e. tonsillotomy and adenotonsillotomy, were predictors of not bleeding. Dissection and hemostatic techniques also seem to be important predictors. In the literature, an association between the amount of warm techniques used and the risk of bleeding has been reported [7]. These patterns also seem to be the case in our material. Of note, the use of diathermy scissors was not a predictor of PTH despite the data-driven subgroup undergoing tonsillectomy with diathermy scissors having the highest rate of PTH. This is likely explained by the statistically “protective” nature of tonsillotomy, which often also is done with diathermy scissors. The presence of inflammation, such as chronic tonsillitis and peritonsillar abscesses, has also been considered a risk factor for bleeding [20]. However, we found that the indication for surgery did not influence the predictions, and that abscess tonsillectomy was predictive of not bleeding.

This study has important strengths. The sample size is large, and there is nationwide coverage. We have applied a comprehensive and robust machine learning approach with appropriate test-set evaluation of only the most promising model which improves generalizability. There are also several limitations: First, there might be ascertainment bias in the outcome. Individuals that experience a PTH may be more motivated to return the 30-day questionnaire. Since the response rate is 62.4% there is a risk that the relatively large group of non-responder bleed more rarely, and thus the overall rate of PTH in our material may be overestimated. This is also supported by the relatively higher PTH rates in our material (6.17%) as compared to other large cohorts where the same definition of PTH was used (0.8–3.5%) [4, 5]. In our study, the outcome was self-reported, whereas in the others the outcome was captured through hospital systems. Still, another large study including more than 45,000 pediatric tonsillectomies, and using ICD-9 codes to capture cases of bleeding, found a PTH rate of 11.9% [6]. Any such ascertainment bias may undermine the generalizability of our model. Second, there could be response bias in both the perioperative questionnaire completed by the surgeons and the 30-day questionnaire completed by the patients. Such bias reduces the validity and precision of the outcome and could decrease performance of the models. Third, we could not differentiate between early and late PTH, which could be problematic. Early and late PTH are hypothesized to have slightly different risk factors [7] possibly affecting a model that incorporates both as the outcome. Moreover, it affects the clinical interpretation of the model because etiologies and management of early and late PTH are different. Fourth, even after calibration, the calibration curve show some deviation from perfect calibration. This decreases the confidence of the probability estimates and may limit the utility of the model as a clinical decision-support tool. Finally, the models were only validated internally in the same dataset, which could limit generalizability, especially to other healthcare systems or countries. Future works should aim to evaluate such models in external datasets.

## Conclusion

This study presents the largest-to-date prediction model for PTH which achieved a moderate predictive performance (AUC 0.71). Decision curve analysis indicates modest benefit, which limits its clinical utility. More research is needed to improve the performance of the model and to assess its clinical utility.

## Supplementary Information

Below is the link to the electronic supplementary material.

Supplementary file1 (DOCX 463 KB)

Supplementary file2 (DOCX 35 KB)

## Data Availability

Data used in this study contains personal sensitive information and is not publicly available and will not be shared owing to ethical and regulatory limitations. The data may be made available to qualified personnel upon application to relevant authorities.
