# Supplementary material for: Development and validation of a machine learning model predicting post-tonsillectomy hemorrhage
Source: Eur Arch Otorhinolaryngol. 2026 Jun 1;283(8):5283–91. doi: 10.1007/s00405-026-10344-3 (PMC13407440; doi:10.1007/s00405-026-10344-3)
Supplement: Supplementary file 1 — Supplementary file1 (DOCX 463 KB) [file 405_2026_10344_MOESM1_ESM.docx]

SUPPLEMENTARY MATERIAL

**Supplementary Table 1**. Characteristics of identified data-driven clusters.

**Supplementary Table 2**. Training and validation performance of all evaluated classifiers.

**Supplementary Table 3.** Decision curve analysis net benefits.

**Supplementary Table 4**. Ranking of the features’ contribution from recursive feature elimination.

**Supplementary Figure 1**. Overview of study design and population.

**Supplementary Figure 2**. Recursive feature elimination plot.

**Hyperparameters of best tuned model.**

**Python packages.**

**Supplementary Table 1**. Characteristics of identified data-driven clusters. Age is summarized as the mean of the cluster. All other variables are presented as the fraction of positives. The clustering figure is included below for reference.

| **Characteristic** | **Cluster** | | | | | | | | |
| --- | --- | --- | --- | --- | --- | --- | --- | --- | --- |
|  | **Light green** | **Brown** | **Grey** | **Green** | **Blue** | **Red** | **Orange** | **Purple** | **Pink** |
| **n** | 1,198 | 573 | 572 | 2.420 | 4,780 | 1,056 | 10,721 | 10,073 | 644 |
| **Patient age (mean)** | 23.84 | 27.69 | 9.01 | 13.91 | 4.64 | 11 | 20.17 | 22.08 | 24.7 |
| **Male gender** | 0.44 | 0.55 | 0.52 | 0.5 | 0.42 | 0.43 | 0.61 | 0.68 | 1 |
| **Ind Obstruction** | 0.36 | 0 | 0 | 0.5 | 0.99 | 0.98 | 0.22 | 0 | 0 |
| **Ind recurrent tonsillitis** | 0.17 | 0 | 0 | 0.5 | 0 | 0 | 0 | 0.97 | 0 |
| **Ind peritonsillitis** | 0.04 | 1 | 0 | 0 | 0 | 0 | 0 | 0 | 0 |
| **Ind chronic tonsillitis** | 0.4 | 0 | 1 | 0 | 0 | 0 | 0.78 | 0 | 1 |
| **Ind systemic complication** | 0 | 0 | 0 | 0 | 0 | 0 | 0 | 0 | 0 |
| **Ind other** | 0.02 | 0 | 0 | 0 | 0 | 0.01 | 0 | 0.03 | 0 |
| **Quinsy tonsillectomy** | 0.01 | 0.18 | 0 | 0 | 0 | 0 | 0 | 0 | 0.01 |
| **Postoperative admission** | 0.09 | 0.35 | 0.26 | 0.25 | 0.24 | 0.15 | 0.15 | 0.11 | 0.04 |
| **Reoperation** | 0.02 | 0.01 | 0.01 | 0.02 | 0.01 | 0.02 | 0.02 | 0.01 | 0.01 |
| **Tonsillectomy** | 0.4 | 0.99 | 0 | 0.5 | 0 | 0 | 0.77 | 1 | 1 |
| **Adenotonsillectomy** | 0.15 | 0 | 1 | 0.5 | 0 | 0 | 0.22 | 0 | 0 |
| **Tonsillotomy** | 0.4 | 0 | 0 | 0 | 0 | 1 | 0 | 0 | 0 |
| **Adenotonsillotomy** | 0.04 | 0 | 0 | 0 | 1 | 0 | 0 | 0 | 0 |
| **Cold steel dissection** | 0.37 | 0.94 | 0.97 | 0.85 | 0.33 | 0.17 | 0.87 | 0.83 | 0 |
| **Bipolar diathermy dissection** | 0.01 | 0.1 | 0.09 | 0.07 | 0.02 | 0.02 | 0.08 | 0.05 | 0.01 |
| **Diathermy scissor dissection** | 0.2 | 0.04 | 0.06 | 0.12 | 0.53 | 0.53 | 0.12 | 0.15 | 0.99 |
| **Radiofrequency dissection** | 0.02 | 0.01 | 0.01 | 0.01 | 0.2 | 0.25 | 0.01 | 0.01 | 0.01 |
| **Other “warm” dissection** | 0.44 | 0.01 | 0 | 0 | 0.04 | 0.03 | 0 | 0 | 0 |
| **No hemostasis necessary** | 0.11 | 0.01 | 0.01 | 0.01 | 0.04 | 0.02 | 0.01 | 0.01 | 0 |
| **Only compression for hemostasis** | 0.28 | 0.01 | 0.05 | 0.06 | 0.18 | 0.21 | 0.06 | 0.05 | 0 |
| **Infiltration with LA+Adr prior to dissection** | 0.35 | 0.42 | 0.41 | 0.32 | 0.17 | 0.16 | 0.38 | 0.43 | 0.05 |
| **Bipolar diathermy for hemostasis** | 0.44 | 0.83 | 0.76 | 0.77 | 0.52 | 0.54 | 0.79 | 0.77 | 1 |
| **Radiofrequency for hemostasis** | 0.03 | 0.03 | 0.01 | 0.01 | 0.03 | 0.05 | 0.02 | 0.03 | 0 |
| **Other “warm” hemostasis** | 0.06 | 0.01 | 0.01 | 0 | 0.01 | 0.01 | 0.01 | 0.01 | 0 |
| **Sutures/ties for hemostasis** | 0.03 | 0.04 | 0.06 | 0.06 | 0 | 0.01 | 0.03 | 0.05 | 0 |
| **Postoperative bleeding prior to discharge** | 0 | 0.01 | 0.01 | 0 | 0 | 0 | 0.01 | 0.01 | 0 |
| **PTH** | 0.05 | 0.07 | 0.04 | 0.06 | 0.01 | 0.02 | 0.07 | 0.08 | 0.11 |

LA: local anesthetic; Adr: adrenaline; PTH: post-tonsillectomy hemorrhage.


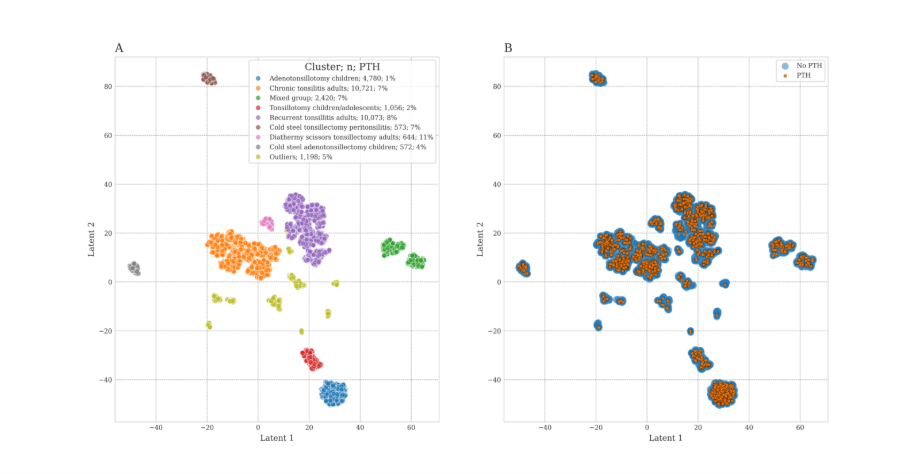


**Supplementary Table 2**. Training and validation performance of all evaluated classifiers.

|  | **Training, mean cross-validation** | | | | | **Validation** | | | | |
| --- | --- | --- | --- | --- | --- | --- | --- | --- | --- | --- |
| **Classifier** | **AUC** | **F1** | **Bal acc** | **Sensitivity** | **Specificity** | **AUC** | **F1** | **Bal acc** | **Sensitivity** | **Specificity** |
| **Logistic regression** | 0.68 | 0.63 | 0.63 | 0.75 | 0.5 | 0.67 | 0.62 | 0.62 | 0.75 | 0.49 |
| **SGD** | 0.67 | 0.59 | 0.61 | 0.77 | 0.46 | 0.66 | 0.57 | 0.6 | 0.78 | 0.43 |
| **Ridge classifier** | 0.68 | 0.62 | 0.63 | 0.77 | 0.49 | 0.67 | 0.61 | 0.62 | 0.76 | 0.48 |
| **Multinomial NB** | 0.65 | 0.58 | 0.61 | 0.76 | 0.45 | 0.64 | 0.59 | 0.61 | 0.78 | 0.45 |
| **Gaussian NB** | 0.64 | 0.5 | 0.59 | 0.83 | 0.36 | 0.64 | 0.52 | 0.59 | 0.8 | 0.38 |
| **KNN** | 0.57 | 0.88 | 0.52 | 0.13 | 0.92 | 0.58 | 0.89 | 0.52 | 0.12 | 0.92 |
| **SVM** | 0.63 | 0.68 | 0.6 | 0.62 | 0.57 | 0.65 | 0.67 | 0.61 | 0.66 | 0.56 |
| **Decision tree** | 0.57 | 0.88 | 0.53 | 0.13 | 0.92 | 0.58 | 0.88 | 0.53 | 0.14 | 0.91 |
| **Random Forest** | 0.61 | 0.88 | 0.53 | 0.13 | 0.92 | 0.62 | 0.88 | 0.53 | 0.16 | 0.9 |
| **Gradient boosting** | 0.68 | 0.75 | 0.61 | 0.55 | 0.68 | 0.68 | 0.75 | 0.64 | 0.61 | 0.66 |
| **Adaboost** | 0.68 | 0.66 | 0.63 | 0.71 | 0.54 | 0.68 | 0.65 | 0.64 | 0.76 | 0.52 |
| **LGBM** | 0.67 | 0.89 | 0.52 | 0.09 | 0.95 | 0.66 | 0.88 | 0.53 | 0.14 | 0.92 |
| **XGB** | 0.64 | 0.88 | 0.53 | 0.15 | 0.92 | 0.63 | 0.87 | 0.52 | 0.14 | 0.89 |

**Supplementary Table 3.** Decision curve analysis net benefits. The net benefit of each strategy (model, bipolar diathermy, “treat all”, and “treat none”) is tabulated against the threshold probabilities.

| **Threshold** | **5%** | **10%** | **15%** | **20%** |
| --- | --- | --- | --- | --- |
| **Model** | 0.03 | 0.01 | 0.0 | 0.0 |
| **Bipolar diathermy** | 0.01 | -0.03 | -0.07 | -0.12 |
| **Treat all** | 0.01 | -0.05 | -0.10 | -0.17 |
| **Treat none** | 0.0 | 0.0 | 0.0 | 0.0 |

**Supplementary Table 4**. Ranking of the features’ contribution from recursive feature elimination. The features ranked as 1 are all included in the optimal model, and their internal ranking is not ordered.

| **Feature** | **Ranking** |
| --- | --- |
| Age | 1 |
| Sex | 1 |
| Repeat surgery | 1 |
| Cold steel dissection | 1 |
| Tonsillotomy | 1 |
| Adenotonsillotomy | 1 |
| Quinsy tonsillectomy | 1 |
| Hemostasis with sutures/ties | 1 |
| Hemostasis with radiofrequency | 1 |
| Hemostasis with other warm technique | 1 |
| Adrenaline infiltration | 1 |
| Hemostasis with bipolar diathermy | 1 |
| No hemostasis needed | 1 |
| Other warm dissection | 1 |
| Dissection with radiofrequency | 1 |
| Dissection with bipolar diathermy | 1 |
| Postoperative hemorrhage prior to discharge | 1 |
| Dissection with diathermy scissors | 2 |
| Only compression for hemostasis | 3 |
| Tonsillectomy | 4 |
| Adenotonsillectomy | 5 |
| Inpatient procedure | 6 |
| Ind. obstruction | 7 |
| Ind. other | 8 |
| Ind. systemic complication | 9 |
| Ind. chronic tonsillitis | 10 |
| Ind. peritonsillitis | 11 |
| Ind. recurrent tonsillitis | 12 |

**Supplementary Figure 1**. Overview of study design and population.


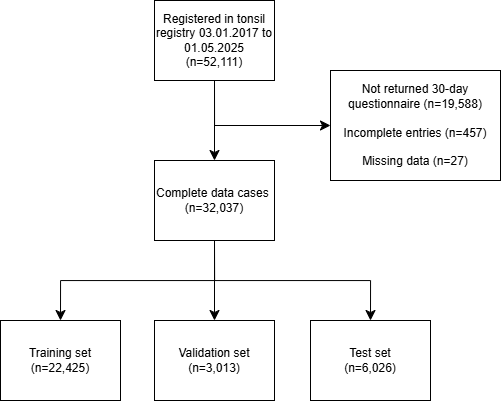


**Supplementary Figure 2**. Recursive feature elimination plot. The optimal number of features is 15. after which inclusion of additional features leads to a plateauing in performance.


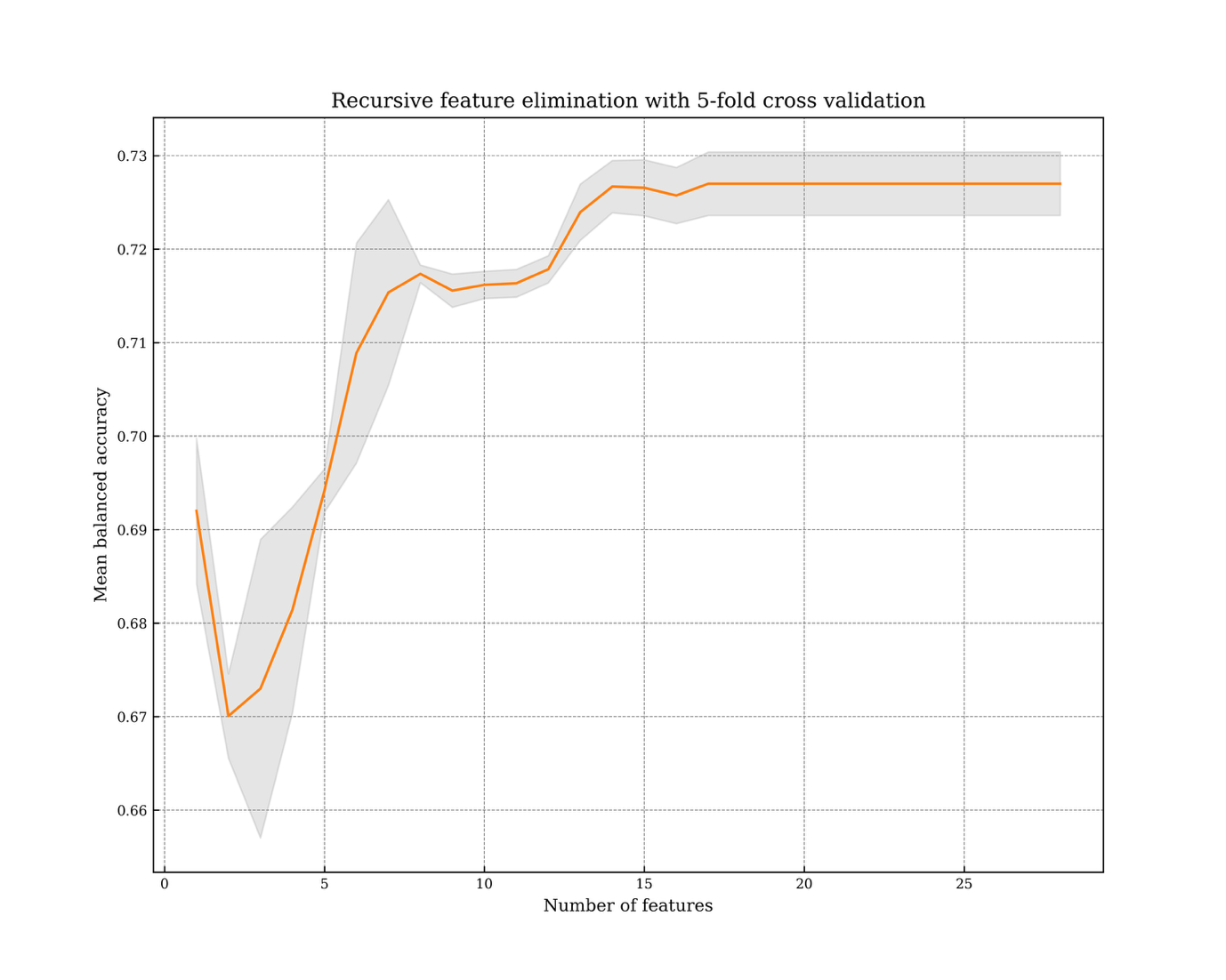


**Hyperparameters of best tuned model.** The best model during training and cross-validation was the AdaBoost Classifier. The hyperparameters were tuned using a Bayesian Search strategy with cross-validation with 50 iterations. The age feature was preprocessed with a MinMaxScaler, and the data was resampled with ADASYN.

‘steps’: [

(‘preprocessor’, ColumnTransformer(remainder=’passthrough’, transformers=[(‘num’, MinMaxScaler(), [‘PatientAge’])])).

(‘resample’, ADASYN(n_neighbors=3, random_state=42, sampling_strategy=1)),

(‘clf’, AdaBoostClassifier(learning_rate=1.1613227027804114, n_estimators=155, random_state=42))

]

**Python packages**

Python 3.11

dcurves 1.1.5

imbalance-learn 0.14.0

lightgbm 4.6.0

matplotlib 3.10.6

numpy 2.2.3

openpyxl 3.1.5

pandas 2.3.2

scikit-learn 1.6.1

scikit-optimize 0.10.2

scipy 1.16.2

seaborn 0.13.2

shap 0.48.0

umap-learn 0.5.9

xgboost 3.0.5
